# Kawasaki disease: an epidemiological study in central Italy

**DOI:** 10.1186/s12969-016-0084-6

**Published:** 2016-04-12

**Authors:** Angela Mauro, Marianna Fabi, Monica Da Frè, Paolo Guastaroba, Elena Corinaldesi, Giovanni Battista Calabri, Teresa Giani, Gabriele Simonini, Franca Rusconi, Rolando Cimaz

**Affiliations:** Department of Pediatrics, Second University of Naples, via Luigi De Crecchio, 80138 Naples, Italy; Pediatric Cardiology and Cardiac Surgery, S. Orsola-Malpighi Hospital, University of Bologna, via Pietro Albertoni 15, 40138 Bologna, Italy; Unit of Epidemiology, Regional Health Agency of Tuscany, Villa La Quiete alle Montalve, via Pietro Dazzi 1, 50141 Florence, Italy; Agenzia Sanitaria e Sociale Regionale - Regione Emilia-Romagna, Area Governo Clinico, Viale A. Moro, 21-40127 Bologna, Italy; Department of Pediatrics, Ramazzini Hospital, Via Guido Molinari, 2, 41012 Carpi, Italy; Cardiology Unit, Anna Meyer Children’s Hospital, Department of Pediatrics, University of Firenze, Viale Pieraccini 24, 50139 Florence, Italy; Rheumatology Unit, Anna Meyer Children’s Hospital, Department of Pediatrics, University of Firenze, Viale Pieraccini 24, 50139 Florence, Italy; Epidemiology Unit, Anna Meyer Children’s University Hospital, Viale Pieraccini 24, 50139 Florence, Italy

**Keywords:** Kawasaki disease, Epidemiology, ICD codes, Incidence, Caucasians

## Abstract

**Background:**

Kawasaki disease (KD) is a systemic vasculitis with an acute and self-limited course. The incidence of KD differs widely among ethnic groups and is higher in the Asian population. In Italy, no recent data are available. Our purpose is to define the epidemiology of Kawasaki disease in the years 2008–2013 in children aged < 14 years in the Italian regions of Tuscany and Emilia Romagna through administrative data.

**Methods:**

We studied the epidemiology of KD in the years 2008–2013 in children 0–14 years old resident in Tuscany and in Emilia Romagna regions using hospital ICD-9 discharge codes with a thorough data cleaning for duplicates.

**Results:**

The distribution of the KD patients across ages was similar for the two regions with a peak in the second year of life. When considering data of the two regions together, the rate of incidence was 17.6 for 100,000 children under 5 years. For both Regions the incidence rose slightly during the study period and had a seasonal distribution, with higher incidence in spring and winter.

**Conclusion:**

This is the first Italian study performed through the use of administrative data. Figures are in line but slightly higher than those published in other European countries.

## Background

Kawasaki disease (KD) is the second most common childhood vasculitis, after Henoch-Schonlein purpura, and predominantly affects children < 5 years of age [1, 2]. In view of the potential severity of the illness, [3] of its possible genetic background, and its unknown etiology, it is important to obtain epidemiological data in different populations. The incidence of KD differs widely among ethnic groups and is higher in the Asian populations (in Japan 239.6/100,000 children < 5 years of age per year) than in Europe, where the incidence ranges from 4.9 to 15.2/100,000/year in the same age group [4, 5]. In Italy, the actual incidence of KD is not known since there is not a national surveillance program, while experiences of individual centers that described the clinical course of the disease in relatively small cohorts of patients have been published more than 10 years ago [6, 7].

The aim of the present study is therefore to define the epidemiology of KD in the years 2008–2013 in children aged < 14 years in Emilia Romagna and Tuscany, two adjacent Regions in Northern and Central Italy, though administrative data collection.

## Methods

The regional hospital discharge records database including all admissions to hospitals for residents in Emilia Romagna and in Tuscany from January 1, 2008 to December 31, 2013 was analyzed. We selected admissions for children and adolescents younger than 15 years of age with KD code ICD9 446.1 as one of the discharge diagnoses. Hospital episodes that were the first admissions with a diagnosis of KD for each subject in the study period were classified as new cases and used as the numerators in the calculation of incidence rates. For these cases we also checked if there were other hospital admissions in the previous 7 years (2001–2007). Data items on each record included the patient’s age, sex, local authority district of residence, date of admission, and all the diagnoses recorded at discharge from hospital. Denominator data were obtained from the national institute of statistics (ISTAT). A simple moving three-year average was used in order to calculate incidence rates during the study period, to smooth out short-term fluctuations.

The study was carried out in compliance with the Italian law on privacy (Art. 20–21, DL 196/2003). Data were anonymized at the regional statistical offices where a unique identifier was assigned to each patient. This identifier does not allow tracing the patient’s identity and other sensitive data. When anonymized administrative data are used to inform health care planning activities, studies are exempt from notification to the Ethics Committee.

## Results

**Fig. 1:**
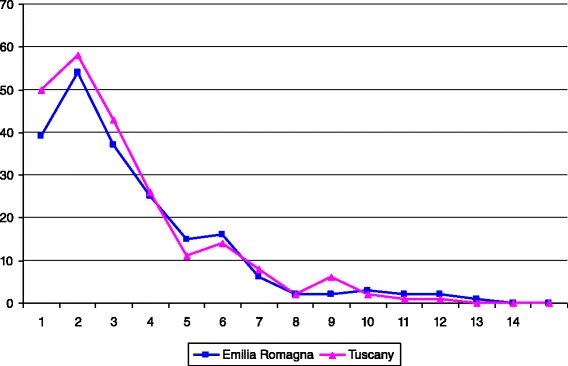
Age distribution of first hospital admissions for KD in children aged 0–14 years by region

During the 6 year period of this study, a total of 426 first hospital admissions for KD in patients younger than 15 years were recorded in the registries from Tuscany (222 cases) and Emilia Romagna (204 cases). The distribution of the KD patients across ages was similar for the two regions with a peak in the second year of life (Fig. 1). All but 57 cases were caucasians.

**Fig. 2:**
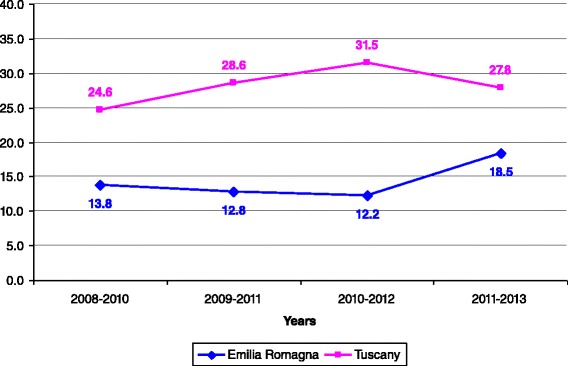
Incidence rates of KD for 100,000 children aged <1 year

**Fig. 3:**
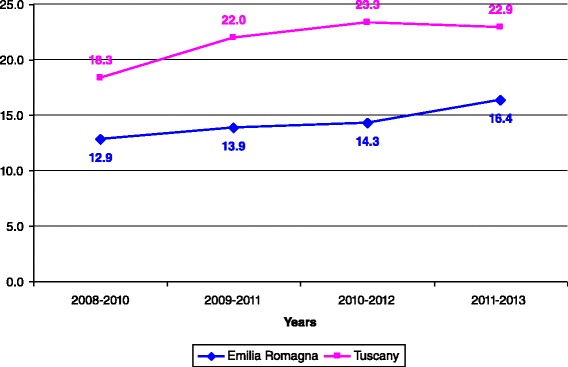
Incidence rate of KD for 100,000 children aged 0 < 5 years

**Fig. 4:**
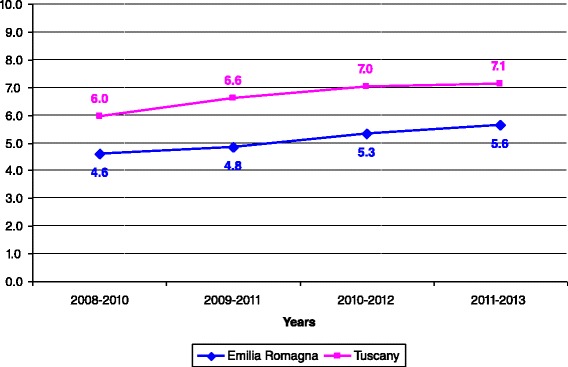
Incidence rate of KD for 100,000 children aged 1–14 years

**Fig. 5:**
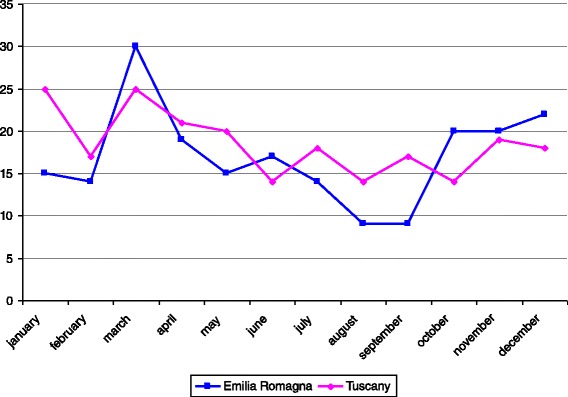
Seasonal distribution of first admission for KD in the two regions during the study period

Incidence rates per 100,000 children aged <1 year, < 5 years, and 1–14 years are reported in Figs. 2, 3, 4, respectively. For all the age classes considered incidence of KD was higher in Tuscany than in Emilia Romagna and slightly rose during the study period. Male patients comprised 62 and 58 % of the total KD number in Emilia Romagna and Tuscany, respectively. The seasonal distribution of KD for all years considered together, is shown in Fig. 5, and showed a nadir in the summer months (June- September).

Twenty-two patients (5.2 %) had at least one cardiac complication, according to ICD9 discharge diagnosis. Coronary artery abnormalities (aneurysms or dilatations) were coded in half of them (*n* = 11), while pericardial involvement was documented in 8, myocardial involvement in 4, and cardiac valve disease in one. Note that the total exceeds 100 % since a few patients had more than one complication.

## Discussion

We described the first epidemiological Italian study performed through the use of administrative data, which evaluates the incidence of Kawasaki disease in two adjacent Italian regions, Emilia Romagna and Tuscany. As expected, the observed incidence of KD is much lower than figures observed in Japan and in other Asian countries.

In Europe, the epidemiology of Kawasaki disease has been described in only few studies and the incidence in children < 5 years of age ranges from 3.6 to 15.2/100,000.

In Denmark, Fischer et al. in a retrospective study describe the epidemiological features of KD, using hospital discharge data codes during the years 1981–2004. The incidence of the diseases increased yearly, being 1.2 per 100,000 between 1981 and 1986 and 4.9 per 100,000 in the period 1999–2004 [8]. Better disease ascertainment is probably the main reason of this increase.

Scandinavian countries have an efficient centralized reporting system for all hospitalized patients, and it is therefore possible to have an accurate description of KD epidemiology. Salo et al. have studied the incidence rate of KD in Finland, Norway and Sweden. In the years 1999–2009 KD cases recorded in the registries from these northern European countries were 1390 [9], being higher in Finland. Considering the same reporting system and similar genetic background, these differences in figures in populations of the same area have yet to be explained. Ascertainment bias could be one possibility, the main author of this study being Finnish, with a considerable interest for the disease and hence with possible better diagnostic capabilities for a disease which is recognized mainly by clinical skills. Of note, with regard to total population our study includes only two regions in Italy, but their total population (8.1 million people) exceeds that of Finland (5.4 millions) and of Norway (5.1 millions).

A few studies estimated the incidence of KD in UK. Harnden et al. using population census data and linked hospital admissions, compared KD admission rates in different geographic areas, with differences in the ethnic composition and degree of urbanization [10]. Gardner-Medwin et al. described the incidence of Henoch-Schonlein purpura and KD in the years 1996–1999 in West Midlands, using questionnaires sent to 321 consultants and 2860 family doctors. They reported 586 cases of vasculitides (463 Henoch-Schonlein purpura, 73 Kawasaki disease, 29 Systemic Lupus erythematosus, 14 Juvenile Dermatomyositis and 8 primary systemic vasculitis) [11]. These differences in figures might be due to different genetic background and different methods of detection (active surveillance vs administrative data). In Ireland, Lynch et al. described the epidemiologic characteristics of KD using hospital discharge records in the period 1996–2000 [12], with a higher incidence when compared to England.

In the Netherlands, Tacke et al. have determined the incidence of KD and evaluated demographic characteristics, treatment and cardiac outcome between 2008 and 2012, using the data of the National Dutch Pediatric Surveillance Unit. They included in their study 341 cases of KD, and showed that annual incidence was 5.8 per 100,000 children aged < 5 years and 8.7 per 100,000 children aged < 1 year [13].

In France, the incidence of KD is not well established. A first study performed with a prospective survey of pediatric departments has been published by Borderon et al. in 1995, and estimated an incidence of 5/100,000 children aged < 5 years [14]. Subsequently, Heuclin et al. described the incidence rates of KD in northern France (involving all pediatric departments in 18 hospitals in Northern France), from September 2005 to August 2006, using the new criteria from the “American Heart Association” and included patients with complete, incomplete and uncertain KD that responded to treatment [15]. Results showed that the incidence of KD in northern France was 9/100,000 children aged < 5 years Kawanet is a recent epidemiological database of KD in France, and was collected by active surveillance. It includes 468 cases from 65 centers (Complete KD and incomplete KD) [16]; however, its design is not suitable to define epidemiological data since these were collected by questionnaire and only a relatively small percentage of the pediatric centers responded to the survey. Finally, a recent study was performed in Tyrol, Austria, which described clinical features of KD in 32 patients (75 % complete, 25 % incomplete KD) over a 10-year period [17]. Again, no incidence data could be drawn in this study.

As for the present study, it has been performed only in two Italian regions, but their total population (8.1 million) exceeds that of several European countries. Our data are in agreement with others from Caucasian populations, with only slightly higher figures.

Data from Australia and New Zealand mirror incidence rates of European- Caucasian populations. In fact, the incidence of KD was 9.34 per 100,000 in children aged <5 years in the years 2000–2009 in Australia [18] and 8 per 100,000 children aged < 5 years in New Zealand during the years 2001–2002 [19].

As mentioned, the highest incidence of KD is found in Asian countries. Makino et al. have described the most recent epidemiological features of KD in Japan. In this study they reported in over two years 26,691 cases of KD and the average annual incidence was 264.8\100,000 children aged < 5 years old in 2012 [20]. Korea reports the second highest incidence of KD in the world. Kim at al. reported the data from the 7th nationwide survey of KD in South Korea. As in Japan, the annual incidence of disease increased over time and was estimated to be 134.4\100,000 children aged < 5 years old in 2011 [21]. Huang et al. have described epidemiological features of KD in Taiwan using data collected from Taiwan National database between the years 2003–2006. In this study, the average annual incidence was 69/100,000 children aged < 5 years [22]. In other Asian countries (China and India) the incidence of KD seems lower than Japan, but there are no accurate nationwide data on trends in KD incidence [23]. In our study most of the cases were caucasians, hence we are not able to compare incidence data among different ethnic groups.

In the United States the incidence varies widely by race and ethnicity and has consistently been higher among Asians and Pacific Islanders. These racial differences show that genetic factors play a major role. The incidence in the US of KD between the years 1997–2007 per 100,000 children aged < 5 years old were 20.8 in 2006, 19.6 in 2003, 17.1 in 2000 and 17.5 in 1997 [24].

The difference between studies can be partly explained by different ascertainment methods; however, a recent Canadian study showed that active surveillance results do not differ substantially from those derived from administrative data [25].

With regard to seasonal peaks, in Japan, Korea and Taiwan the disease seems more prevalent in summer and winter, while in Europe and USA seasonal peaks are observed in winter and spring [26]. Our figures seem in agreement with the latter, but numbers are too small to draw firm conclusions.

Burns et al. have studied a global seasonal patterns of KD. They analyzed the seasonal distribution of KD in the years 1970–2012 from 25 countries. In the extra-tropical latitudes of the Northern hemisphere, the cases of KD cases were higher from January to March than from August to October. On the contrary, in the tropics and the Southern Hemisphere extra-tropics the maximum incidence of KD was between May-June and was lower between February, March and October. These results support the hypothesis that an environmental trigger might have a role in determining the differing seasonality of KD cases worldwide [27]. One mechanism might explain the hemispheric seasonal distributions of KD: the recent observation that large scale tropospheric wind patterns could be associated with fluctuations in KD cases. Rodò et al. hypothesized a possible KD agent spread by means of direct airborne sampling conducted over Japan, followed by a detailed analysis of nucleic acids extracted from aerosolized atmospheric samples trapped on filters collected at selected altitudes [28]. Mountains that separate regions could partially explain different incidence figures in adjacent areas (such as the two regions in our study).

Some limitations are present in the present study. Administrative data may show partial or incomplete information that could affect the results of analyses because it is not possible to evaluate if the diagnosis is correct or not. For this reason it is not possible to understand if the increase of incidence in the years, compared to the past, is due to an improvement in the diagnostic capability or a real increase of the incidence.

## Conclusion

In conclusion, our results derived from administrative discharge data are the first to describe the distribution of KD in two adjacent Regions in Northern and Central Italy, and seem in line with those of other Caucasian populations.

## Abbreviation

KD: Kawasaki disease
